# Potentiating Oncolytic Virus-Induced Immune-Mediated Tumor Cell Killing Using Histone Deacetylase Inhibition

**DOI:** 10.1016/j.ymthe.2019.04.008

**Published:** 2019-04-14

**Authors:** Victoria A. Jennings, Gina B. Scott, Ailsa M.S. Rose, Karen J. Scott, Gemma Migneco, Brian Keller, Katrina Reilly, Oliver Donnelly, Howard Peach, Donald Dewar, Kevin J. Harrington, Hardev Pandha, Adel Samson, Richard G. Vile, Alan A. Melcher, Fiona Errington-Mais

**Affiliations:** 1 The Institute of Cancer Research, Division of Radiotherapy and Imaging, Chester Beatty Laboratories, London SW3 6JB, UK; 2 Section of Infection and Immunity, Leeds Institute of Medical Research, University of Leeds, Beckett Street, Leeds LS9 7TF, UK; 3 Ottawa Hospital Research Institute, Ottawa, ON, Canada; 4 St James’s University Hospital, Leeds LS9 7TF, UK; 5 Leggett Building, Faculty of Health and Medical Sciences, University of Surrey, Guildford GU2 7WG, UK; 6 Mayo Clinic, Rochester, MN 55905, USA

**Keywords:** oncolytic virus, herpes simplex virus, HSV,cancer immunotherapy, histone deacetylase inhibitor, valproic acid, VPA

## Abstract

A clinical oncolytic herpes simplex virus (HSV) encoding granulocyte-macrophage colony-stimulating factor (GM-CSF), talimogene laherparepvec, causes regression of injected and non-injected melanoma lesions in patients and is now licensed for clinical use in advanced melanoma. To date, limited data are available regarding the mechanisms of human anti-tumor immune priming, an improved understanding of which could inform the development of future combination strategies with improved efficacy. This study addressed direct oncolysis and innate and adaptive human immune-mediated effects of a closely related HSV encoding GM-CSF (HSV^GM-CSF^) alone and in combination with histone deacetylase inhibition. We found that HSV^GM-CSF^ supported activation of anti-melanoma immunity via monocyte-mediated type I interferon production, which activates NK cells, and viral maturation of immature dendritic cells (iDCs) into potent antigen-presenting cells for cytotoxic T lymphocyte (CTL) priming. Addition of the histone deacetylase inhibitor valproic acid (VPA) to HSV^GM-CSF^ treatment of tumor cells increased viral replication, viral GM-CSF production, and oncolysis and augmented the development of anti-tumor immunity. Mechanistically, VPA increased expression of activating ligands for NK cell recognition and induced expression of tumor-associated antigens, supporting innate NK cell killing and CTL priming. These data support the clinical combination of talimogene laherparepvec with histone deacetylase inhibition to enhance oncolysis and anti-tumor immunity.

## Teaser

Jennings et al., demonstrate that oncolytic HSV^GM-CSF^ activates NK cells and matures immature dendritic cells to promote cytotoxic T lymphocyte (CTL) priming. Moreover, they report that histone deacetylase inhibition can augment innate and adaptive anti-tumor immune responses through increased expression of NK cell-activating ligands and tumor-associated antigens.

## Introduction

Oncolytic viruses (OVs) are naturally occurring or genetically engineered viruses with specific anti-tumor effects mediated both by direct oncolysis and activation of innate and adaptive anti-tumor immunity. A range of OVs has progressed to clinical studies, and some viruses (e.g., herpes simplex, vaccinia, and reovirus) have reached evaluation in randomized clinical trials.^1^ The most clinically advanced agent (approved for use in the United States, Europe, and Australasia) is a genetically modified double-stranded DNA herpes simplex virus (HSV; JS-1 strain) called talimogene laherparepvec (T-Vec). This virus has been rendered tumor-selective through functional deletion of ICP34.5; further deletion of ICP47 enhances antigen presentation and brings the viral US11 gene under the control of the ICP47 immediate-early promoter, enhancing tumor-selective replication.^2^ In addition, the ICP34.5 gene has been replaced with a cassette encoding human GM-CSF to facilitate priming of an anti-tumor immune response,^3^ and an initial clinical report has confirmed that the virus can convert an immunologically suppressive “cold” tumor microenvironment (TME) into an immune-activating “hot” milieu.^4^ Hence, T-Vec has a dual mode of action, causing direct tumor cell lysis and bystander activation of an anti-tumor immune response.

Following a phase I study demonstrating acceptable toxicity,^5^ phase II testing of intratumoral T-Vec in patients with advanced melanoma resulted in a 26% response rate, with durable responses observed in both injected and uninjected lesions.^6^ Distant responses suggested generation of anti-tumor immunity, which was consistent with experiments showing an increase in melanoma-associated antigen-specific T cells and a decrease in suppressive T cells in tumors after treatment.^7^ These encouraging clinical trial data led to a randomized phase III study in melanoma, comparing intratumoral injection against subcutaneous GM-CSF,^8^ which achieved its primary endpoint of durable response rate.^9^ Early clinical trials also demonstrated that T-Vec was present in the blood and uninjected lymph nodes as well as in the injected tumor,^5^, ^10^ suggesting that viral systemic immune activation and priming instigated within the directly targeted TME may play an additional role in bystander immune-mediated therapy. Randomized trials testing a combination of T-Vec with immune checkpoint inhibitors in melanoma have now been completed, with early results showing significant promise.^4^, ^11^

Despite this clinical progress, pre-clinical data on the mechanisms responsible for the therapeutic potential of T-Vec are relatively limited, and further information would inform the development of future combination therapies. One promising strategy is to combine OVs with histone deacetylase (HDAC) inhibitors (HDACIs), which regulate chromatin structure and gene transcription. Histone acetylation is regulated by the opposing actions of histone acetyltransferases (HATs), which mediate the acetylation of histone residues allowing gene transcription, whereas HDACs remove acetyl groups, allowing the negatively charged DNA to bind the nucleosome, acting as transcriptional repressors. HDACs are classified into four different subclasses based on their sequence homology and structural similarity: class I HDACs (HDAC1, HDAC2, HDAC3, and HDAC8), class II HDACs (HDAC4, HDAC5, HDAC7, HDAC9, and HDAC10), class III HDACs (sirtuins), and a class IV HDAC (HDAC11). High expression of class I and II HDACs has been associated with poor patient outcome, and HDACIs have been developed as anticancer agents. HDAICs induce a diverse range of biological responses in tumors, including apoptosis, suppressed proliferation of malignant cells, inhibition of angiogenesis, and immunomodulation.^12^, ^13^, ^14^, ^15^ Specifically, in terms of immunomodulation, HDACIs have been reported to increase antigen presentation (through modulation of major histocompatibility complex [MHC] molecules), increase T cell recognition, increase natural killer (NK) cell-activating ligand expression and NK cell-mediated killing, increase ICAM-1 expression to promote leukocyte infiltration, enhance immunological synapse formation between T cells and antigen-presenting cells (APCs), and decrease levels of regulatory T cells (Tregs).^13^

Clinically, HDACIs have gained Food and Drug Administration (FDA) approval for the treatment of cancer, including vorinostat and FK228 for the treatment of cutaneous T cell lymphoma (CTCL), belinostat for the treatment of peripheral T cell lymphoma (PTCL), and panobinostat, in combination with bortezomib and dexamethasone for the treatment of multiple myeloma. Although most HDACIs have been approved for the treatment of hematological malignancies, numerous studies and clinical trials have examined their activity against solid malignancies, such as ovarian and breast cancer.^14^ Valproic acid (VPA), an anticonvulsant agent and more recently described HDACI with specificity toward class I and class IIa HDACs, has also been reported to display anticancer properties through induction of cell differentiation, inhibition of cell proliferation, and/or altered immunogenicity. VPA acts by directly inhibiting HDACs but also induces proteasomal degradation of HDAC2, exerting its anticancer properties by both transcription-dependent and transcription-independent mechanisms. Currently, VPA is not FDA-approved for the treatment of cancer; however, it has been studied extensively in pre-clinical models and has reached phase III clinical testing for cervical and ovarian cancer.^15^ Pivotally, VPA is an approved treatment option for epilepsy, bipolar disorder, and migraine prevention and has a well-established safety profile derived from decades of clinical use. Moreover, VPA is a cost-effective treatment option in comparison with newer HDACIs, making re-purposing this agent an attractive option for the treatment of cancer.^15^

HDACIs, including VPA, have been successfully tested in combination with OVs,^16^, ^17^ and a range of synergistic mechanisms have been identified, including (1) suppression of anti-viral interferon (IFN)-responsive gene transcription, leading to increased viral replication, spread, and oncolysis/apoptosis; (2) induction of nuclear factor κB (NF-κB) signaling, resulting in NF-κB-dependent autophagy; (3) increased viral entry receptor expression and viral entry; (4) abrogation of innate immune-mediated viral clearance; and (5) enhancement of adaptive anti-tumor immune responses through enhanced CD8 T cell and macrophage infiltration and decreased Tregs, with appropriate combination scheduling.^18^, ^19^, ^20^ To date, VPA has been reported to enhance HSV and parvovirus replication in cancer cells,^16^, ^21^ but its efficacy in combination with HSV for melanoma has not been described; moreover, the effect of VPA in OV-induced human immunotherapy remains unknown. Here we describe the use of clinically relevant human models^22^, ^23^, ^24^, ^25^, ^26^ to explore oncolytic HSV^GM-CSF^ and VPA immune co-operation to support the development of anti-tumor immune responses against human melanoma.

## Results

### HSV^GM-CSF^ Induces Innate and Adaptive Anti-tumor Immunity

**Figure 1:**
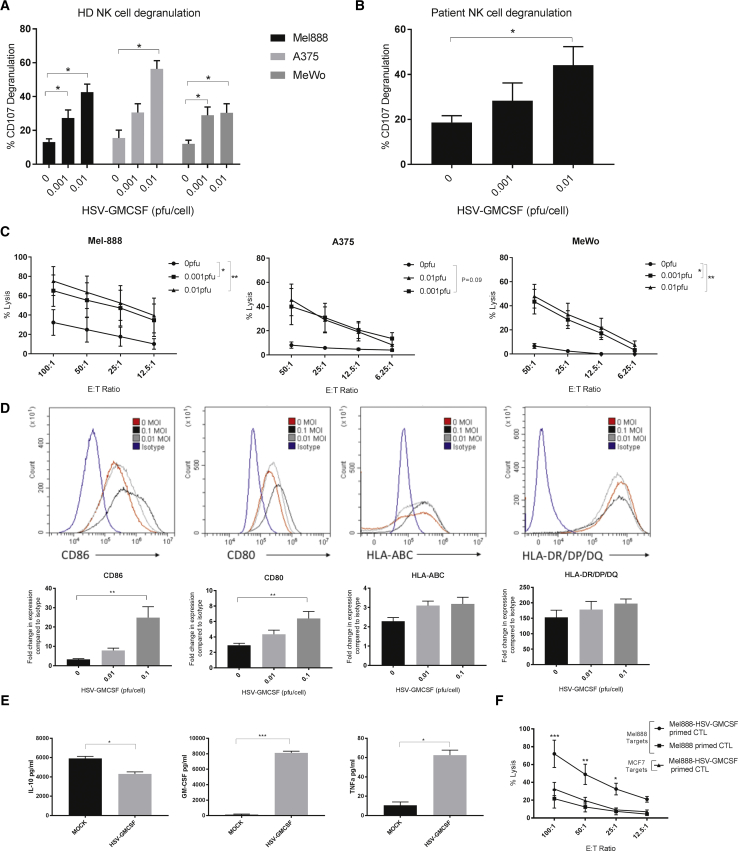
HSV^GM-CSF^ Induces Innate and Adaptive Anti-tumor Immunity (A) Healthy donor PBMC (with or without HSV^GM-CSF^ treatment) were co-cultured with melanoma targets, and NK cell (CD56^+^/CD3^−^) CD107 degranulation was determined by flow cytometry. The mean percentage of NK cells degranulating after co-culture with MEL888, A375, and MeWo tumor cell targets + SEM is shown (at least n = 4). (B) PBMCs from melanoma patients with metastatic disease (with or without HSV^GM-CSF^ treatment) were co-cultured with melanoma targets (MEL888 cells), and NK cell (CD56^+^/CD3^−^) CD107 degranulation was determined by flow cytometry. The mean percentage of NK cells expressing CD107 + SEM is shown (n = 4). (C) Healthy donor PBMCs (with or without HSV^GM-CSF^) were co-cultured with MEL888, A375, and MeWo cell targets, and the percentage of tumor cell lysis was determined by ^51^Cr release. The graph shows the mean of at least three experiments ± SEM. (D) Immature dendritic cells were treated with or without HSV^GM-CSF^ for 48 h, and cell surface expression of CD86, CD80, HLA-ABC, and HLA-DR/DP/DQ was determined by flow cytometry. Representative histograms (top panel) and the mean fold increase in expression compared with isotype controls + SEM (bottom panel) are shown (n = 4). (E) Supernatants from melanoma cells treated with or without HSV^GM-CSF^ and co-cultured with iDCs were collected, and the concentrations of GM-CSF, IL-10, and TNF-α were determined by ELISA. The graph shows the mean + SEM (n = 3). (F) MEL888 cells were either left untreated (Mel888-primed CTLs) or treated with 0.1 PFUs/cell HSV^GM-CSF^ (Mel888+HSV-GM-CSF-primed CTLs) and cultured with iDCs for 24 h before non-adherent cells were removed and cultured with autologous PBMCs. CTLs were re-stimulated once (as appropriate) and then used in 4-h ^51^Cr release assays against MEL888 (relevant) or MCF-7 (irrelevant) targets. The graph shows the mean percentage of tumor cell death ± SEM (n = 3). Statistical significance is denoted by *p < 0.05, **p < 0.01, and p*** < 0.005.

We previously developed *in vitro* pre-clinical assays to test the potential of OVs to support the activation of human innate (dendritic cells [DCs] and NK cells) and adaptive (cytotoxic T lymphocytes [CTLs]) anti-tumor immunity.^22^, ^24^, ^25^, ^26^ To initially address the immunogenicity of HSV^GM-CSF^, we pulsed the virus onto peripheral blood mononuclear cells (PBMCs) taken from healthy donors and melanoma patients and examined activation of NK cells. Addition of HSV^GM-CSF^ induced NK cell degranulation (release of cytotoxic granules) in both healthy donor (Figure 1A) and patient samples (Figure 1B) upon co-culture with melanoma cell targets, as determined by increased expression of CD107 on NK cells. Importantly, HSV^GM-CSF^-induced NK cell degranulation correlated with increased lysis of melanoma cell targets (Figure 1C). To confirm that NK cells were responsible for melanoma target cell death, in the context of PBMCs, we have shown that (1) depletion of NK cells from PBMCs significantly reduced killing of MEL888 cells (Figure S1A) and (2) that killing was mediated by perforin and granzyme (pivotal components of NK cell cytotoxic granules) because cell lysis was abrogated by EGTA, a calcium chelator that prevents the activity of calcium-dependent perforin (Figure S1B).

Having shown previously that OVs can activate DCs, pivotal APCs that bridge both the innate and adaptive arms of the immune system,^22^ we investigated the effect of HSV^GM-CSF^ on the DC antigen-presenting machinery (MHC class I and II) and co-stimulatory molecules (CD80 and CD86). We found that HSV^GM-CSF^ induced maturation of immature DCs (iDCs), causing significant upregulation of CD80 and CD86 and retention of MHC class I and II (Figure 1D) without significantly decreasing cell viability (data not shown). Next, to determine whether iDCs were infected with HSV^GM-CSF^ and whether this was required to induce DC maturation, we treated iDCs with GFP-expressing HSV (0.01 and 0.1 plaque-forming units [PFUs]/cell) and examined CD86 expression in GFP-positive (HSV-infected) and GFP-negative (non-infected) DCs. At the highest MOI, approximately 10% of DCs were GFP-positive, and in accordance with this, a 10% loss in viability was observed, demonstrating that DCs were indeed permissive to HSV infection and subsequent cell death (data not shown). However, importantly, CD86 upregulation was observed in GFP-negative DCs, suggesting an indirect mechanism of DC maturation, potentially mediated by cytokine release (data not shown).

Furthermore, co-culture of HSV^GM-CSF^-infected tumor cells with iDCs resulted in increased secretion of GM-CSF and tumor necrosis factor alpha (TNF-α), together with decreased production of the immunosuppressive cytokine interleukin-10 (IL-10) (Figure 1E); virus-infected melanoma cells secrete GM-CSF, as expected (Figure 3); therefore, it is most likely that the GM-CSF production is derived from infected tumor cells. However, given that up to 10% of DCs can be infected by HSV, it is also possible that DCs may contribute to GM-CSF production. Moreover, MEL888 cells secrete IL-10, which can be downregulated by OV treatment,^27^ and iDCs produce TNF-α following OV treatment;^28^ therefore, although we have not specifically demonstrated that the changes in IL-10 and TNF-α levels are due to effects on MEL888 cells and iDCs, respectively, we postulate that this is the most likely explanation.

Finally, to assess whether HSV^GM-CSF^-induced DC maturation and changes in the pro-inflammatory cytokine milieu supported adaptive CTL immune priming, we loaded iDCs with HSV^GM-CSF^-infected tumor cells and examined whether tumor-loaded DCs could support the generation of tumor-specific CTLs.^22^, ^24^, ^26^ Figure 1F shows that virus-infected tumor cells supported the generation of melanoma-specific CTLs whereas non-infected tumor cells did not. Taken together, these data show that HSV^GM-CSF^ has the potential to enhance both innate and adaptive anti-tumor immune responses.

### Activation of a Human Innate Immune Response by HSV^GM-CSF^ Is Dependent on Virus-Induced Type I IFN Production

**Figure 2:**
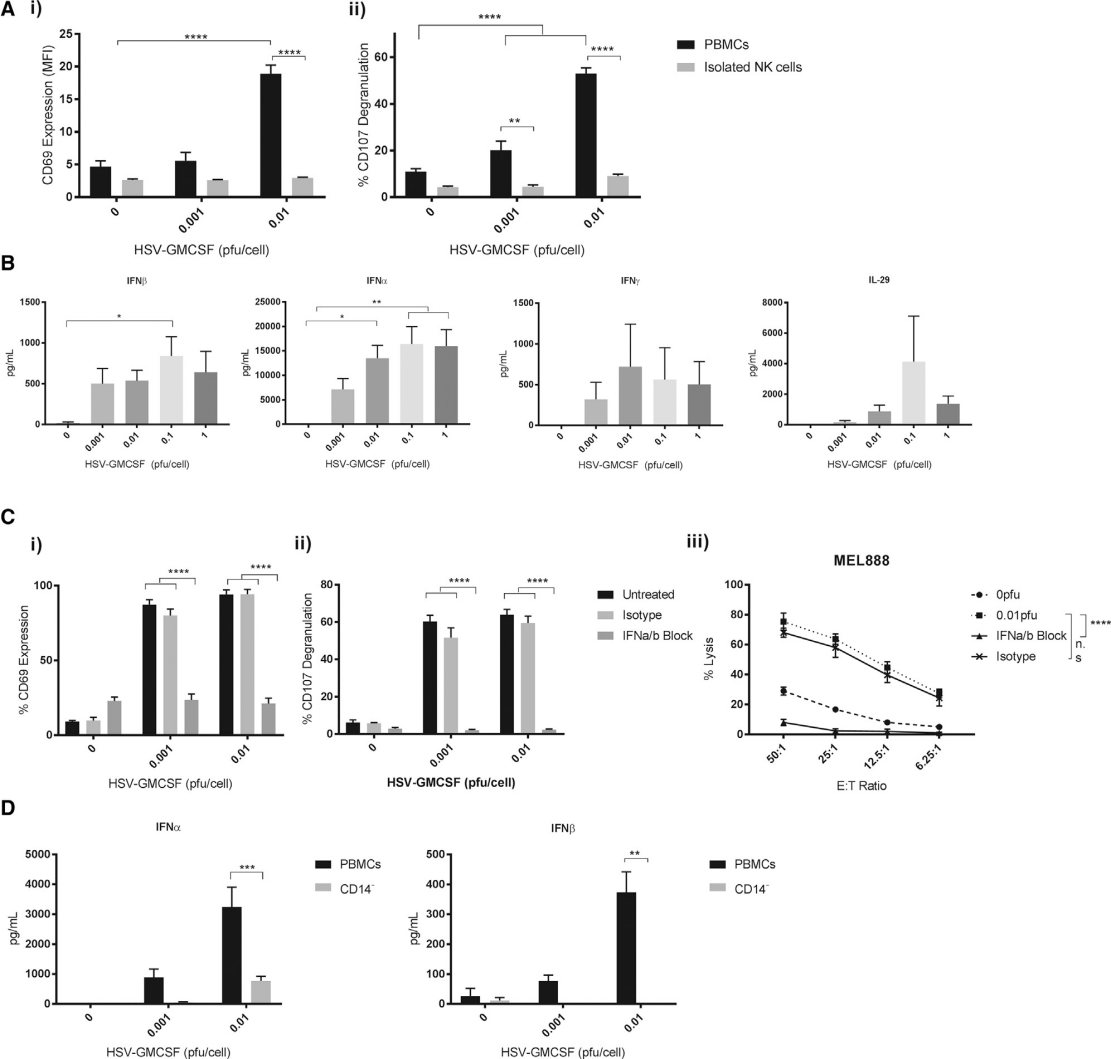
Innate Activation Is Dependent on Type I IFNs and CD14^+^ Monocytes (A) Healthy donor PBMCs or isolated NK cells were treated with HSV^GM-CSF^ for 48 h and NK cell (CD56^+^/CD3^−^) (i) CD69 expression and (ii) CD107 degranulation (following co-culture with melanoma targets) were determined by flow cytometry (n = 4). (B) Healthy donor PBMCs were treated with HSV^GM-CSF^ for 48 h, and production of IFNγ, IFNα, IFNβ, and IL-29 was determined by ELISA. The graph shows the mean of at least four independent experiments + SEM. (C) Healthy donor PBMCs were treated overnight with HSV^GM-CSF^ either alone or in the presence of IFNα/β blocking antibodies or isotype controls before (i) CD69 upregulation on CD56^+^/CD3^−^ NK cells was determined by flow cytometry. The graph shows the average percentage of NK cells expressing CD69 + SEM (n = 3). (ii) PBMCs (with or without IFN blockade and with or without HSV^GM-CSF^) were co-cultured with MEL888 cells, and NK cell CD107 degranulation was determined by flow cytometry. The graph shows the mean percentage of NK cells expressing CD107a/b + SEM (n = 3). (iii) PBMCs (with or without IFN blockade and with or without HSV^GM-CSF^) were co-cultured with MEL888 cells at the indicated E:T ratios, and the percentage of tumor cell lysis was determined by ^51^Cr release. The graph shows the mean percentage lysis ± SEM (n = 3). (D) IFNα/β production from whole PBMCs or CD14^+^ monocyte-depleted PBMCs was determined by ELISA. The graph shows the mean + SEM (n = 5). Statistical significance is denoted by *p < 0.05, **p < 0.01, ***p < 0.005, and ****p < 0.0001.

To characterize the mechanisms responsible for innate NK cell activation following HSV^GM-CSF^ treatment, we first examined the ability of HSV^GM-CSF^ to activate isolated NK cells. NK cells isolated from PBMCs and subsequently treated with HSV^GM-CSF^ directly were unable to degranulate against melanoma targets and showed no upregulation of the early activation marker CD69 (Figure 2A). Furthermore, when PBMCs were depleted of CD14^+^ monocytes (shown previously to be central to the immune response induced by an alternative OV, reovirus^24^), we found that HSV^GM-CSF^ treatment did not result in activation of NK cells, as assessed by surface CD69 expression, relative to intact PBMCs, which included monocytes (Figure S2A); additionally, NK cell-mediated killing was also significantly abrogated (Figure S2B). Therefore, these data support a role for monocytes within PBMCs in mediating the activation of NK cells by HSV^GM-CSF^.

Upon further examination, we demonstrated that PBMCs treated with HSV^GM-CSF^ secreted type I, II, and III IFNs (Figure 2B) and that blockade of type I IFNα/β abrogated HSV^GM-CSF^-induced activation of NK cells in terms of CD69 expression (Figure 2Ci), NK cell CD107 degranulation (Figure 2Cii), and cytotoxicity against melanoma cells (Figure 2Ciii). Furthermore, type I IFN production, like NK cell activation, was significantly abrogated when CD14^+^ monocytes were depleted from PBMCs (Figure 2D). Taken together, these data show that the innate response of NK cells following HSV^GM-CSF^ treatment of PBMCs was dependent on type I IFN production and confirmed a role of CD14^+^ monocytes in mediating type I IFN secretion.

### HDAC Inhibition Enhances HSV^GM-CSF^ Replication, Killing, and GM-CSF Production in Melanoma Cells

Having shown that HSV^GM-CSF^ induces innate and adaptive immune responses in our human model systems, we subsequently examined the ability of HDACIs to potentiate HSV^GM-CSF^ efficacy in terms of direct cytotoxicity and HSV^GM-CSF^-induced anti-tumor immunity. First, to investigate the direct cytopathic effects of HSV^GM-CSF^ in the presence or absence of HDACIs, MEL888 cells were pre-treated with a range of HDACIs (VPA, tubastatin, vorinostat, droxinostat, givinostat, and mocetinostat) for 24 h before addition of HSV^GM-CSF^ (at concentrations of up to 2.5 PFUs/cell) for a further 48 h, and cell viability was determined by microculture tetrazolium test (MTT) (Figure S3). These data demonstrated that all HDACIs tested were able to enhance HSV^GM-CSF^ cytotoxicity, although, as expected, the results were variable, and the greatest potentiation was observed for VPA, givinostat, and mocetinostat. Given the long-standing safety profile of VPA and the cost-effective nature of this agent, VPA was selected for further experimentation.

**Figure 3:**
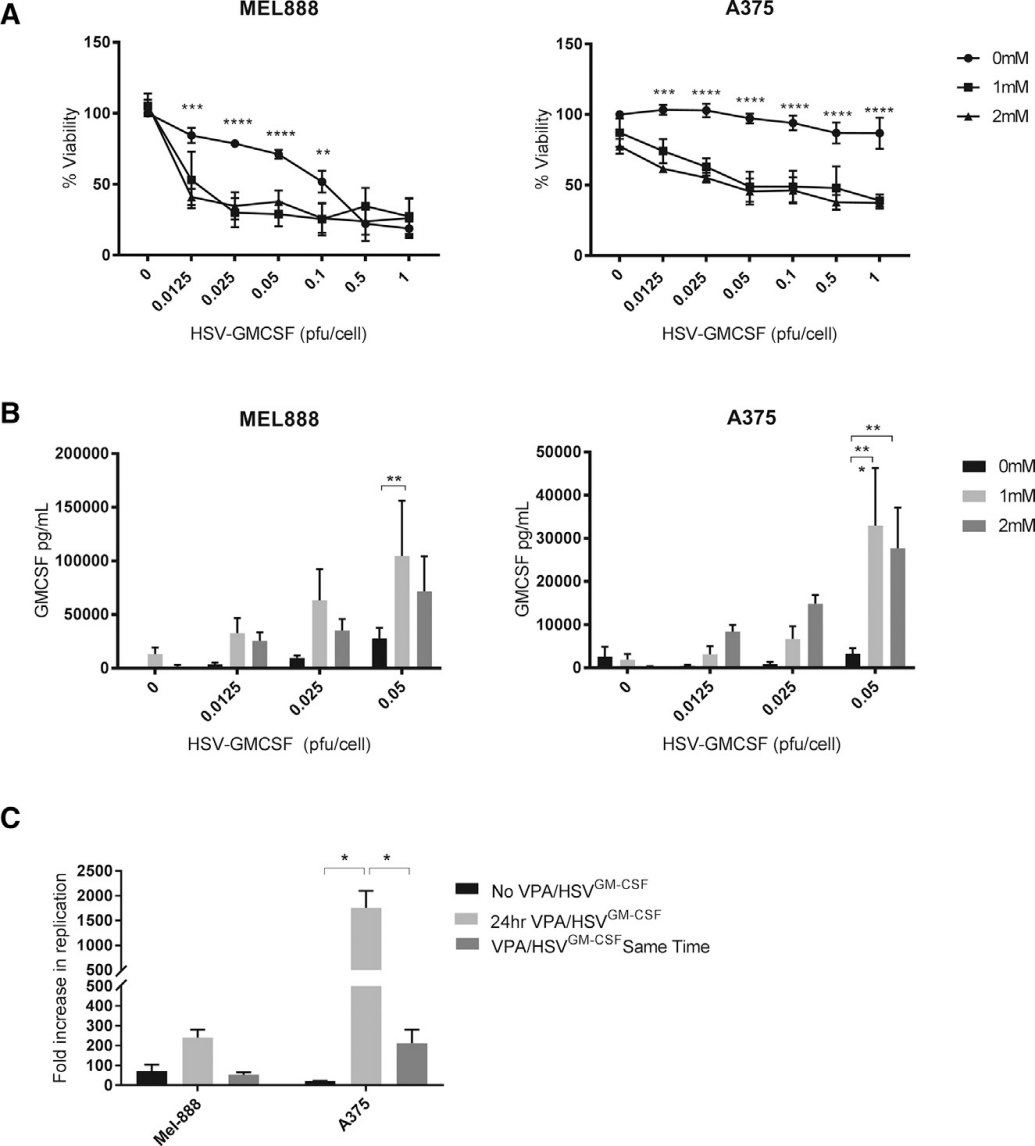
VPA Enhances HSV^GM-CSF^-Induced Cytotoxicity, Viral Replication, and Transgene Expression (A) Melanoma cell lines were seeded and treated with VPA (0, 1, and 2 mM) for 24 h prior to addition of HSV^GM-CSF^ at concentrations ranging from 0 to 1 PFUs/cell. Cells were left for a further 48 h, and cell viability was determined by MTT assay. The graph shows the average cell viability for at least five independent experiments ± SEM. (B) VPA-treated melanoma cells were treated with HSV^GM-CSF^ for 24 h, and GM-CSF production was determined by ELISA. The graph shows the mean + SEM (n = 6). (C) MEL888 or A375 cells were treated with 0.05 PFUs/cell HSV^GM-CSF^ alone, 1 mM VPA for 24 h prior to 0.05 PFUs/cell HSV^GM-CSF^, or 1 mM VPA and 0.05 PFUs/cell HSV^GM-CSF^ simultaneously. Cells were left for 24 h, and the fold increase in HSV^GM-CSF^ replication was determined by plaque assay. The graph shows the mean + SEM (n = 4). Statistical significance is denoted by *p < 0.05, **p < 0.01, p*** < 0.005, and ****p < 0.0001.

Initially, the ability of VPA to potentiate HSV^GM-CSF^ cytotoxicity against a larger panel of melanoma cell lines was examined. All cells lines were susceptible to HSV^GM-CSF^-induced cytotoxicity with variable sensitivity, and VPA significantly increased the direct cytotoxic effect of HSV^GM-CSF^ in all cell lines tested (Figure 3A; Figure S4A). Next, the ability of HSV^GM-CSF^ to induce secretion of GM-CSF and replicate in melanoma cells was determined. GM-CSF was produced upon infection of all cell lines (Figure 3B; Figure S4B), although the levels were lower in A375 cells compared with MEL888 cells (Figure 3A); significantly, VPA increased GM-CSF secretion in both the relatively resistant (A375) and sensitive (MEL888) cell lines (Figure 3B). Additionally, plaque assays confirmed the production of infectious progeny virus and showed that VPA increased HSV^GM-CSF^ replication (Figure 3C), with potentiation by VPA being most evident in A375 cells, which were inherently less permissive to viral replication. In terms of scheduling of the two reagents, and consistent with previous data,^16^ we also found that enhanced HSV^GM-CSF^ replication was dependent on pre-treatment with VPA; otherwise, no increase in viral replication was seen (Figure 3C). Importantly, HSV^GM-CSF^ cytotoxicity against non-neoplastic human foreskin fibroblasts (HFFs) was not enhanced upon combination with VPA (Figure S5), suggesting that VPA would not increase off-target side effects caused by viremia in non-malignant tissue. Collectively, these data confirm that HSV^GM-CSF^ directly infects, kills, and replicates in human melanoma cells, resulting in secretion of GM-CSF, and that addition of VPA potentiates these effects, particularly in cells that are otherwise relatively poorly permissive.

### HDAC Inhibition Augments HSV^GM-CSF^-Induced Innate Anti-tumor Immunity

**Figure 4:**
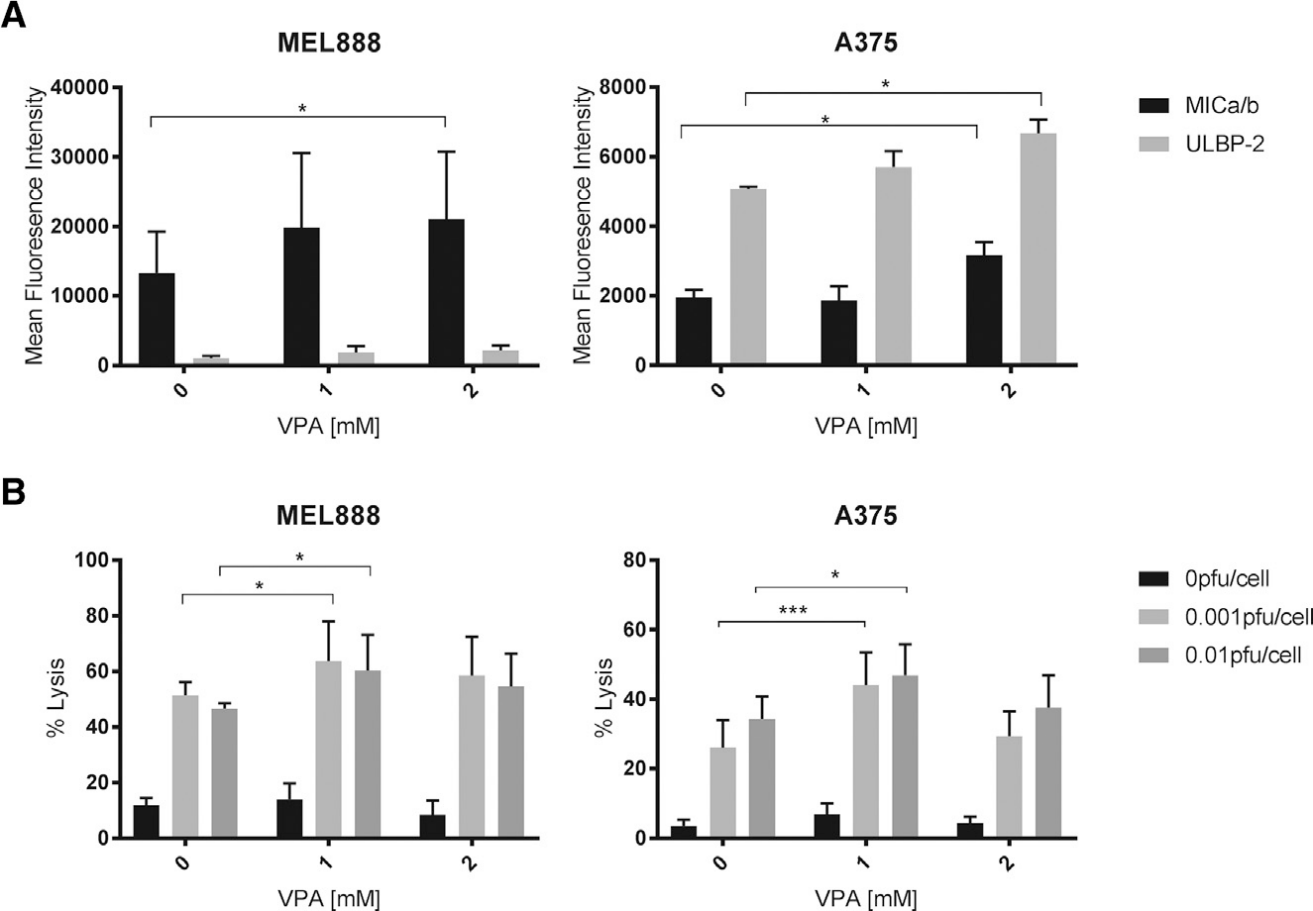
VPA Augments HSV^GM-CSF^ Innate Anti-tumor Immunity (A) Expression of NK ligands (MICA/B and ULBP2/5/6) on the surface of melanoma cells was determined by flow cytometry. Cells were treated with VPA at the indicated doses for 48 h. Mean fluorescence intensity is shown + SEM (n = 3). (B) Healthy donor PBMCs (untreated [0 PFUs] or activated with HSV^GM-CSF^ [0.001 or 0.01 PFUs] overnight) were co-cultured with melanoma cells with or without VPA for 5 h, and the percentage of target cell death was determined by flow cytometry. The graph shows the mean + SEM for at least four independent experiments. Statistical significance is denoted by *p < 0.05, **p < 0.01, and p*** < 0.005.

Having shown that VPA increases killing, replication, and GM-CSF production upon HSV^GM-CSF^ treatment of human melanoma cells, we next tested the effects of VPA on HSV^GM-CSF^-induced innate anti-tumor immunity. To address this, we first tested whether VPA affected the expression of activating NK ligands on human melanoma cells; VPA has been reported previously to upregulate NK ligand expression on acute myeloid leukemia cells *in* vivo.^29^, ^30^ We observed upregulation of the NKG2D ligands MICA/B on MEL888 cells and MICA/B and ULBP2/5/6 on less permissive A375 cells upon treatment with VPA (Figure 4A); similar results were also observed using primary melanoma cells (Figure S6A and data not shown). This suggested that addition of VPA could directly support innate anti-tumor immunity by increasing activating NK cell:tumor target interactions; this was subsequently confirmed because VPA treatment of melanoma cells prior to their co-culture with HSV^GM-CSF^-treated PBMCs caused increased NK cell-mediated tumor cell killing (Figure 4B). Importantly, additional studies have confirmed that alternative HDACIs also upregulate the expression of NK cell-activating ligands on melanoma tumor cells (Figure S6B), suggesting that the effects of VPA were due to HDAC inhibition and not an alternative, HDAC-independent mechanism of action.

Furthermore, in line with previously published data that showed that VPA was only toxic to NK cells at doses greater than 2.5 mM,^31^ we confirmed that pre-treatment of PBMCs with VPA prior to HSV^GM-CSF^ stimulation (e.g., the schedule required for enhanced direct oncolysis) does not inhibit production of type I IFNα from PBMCs (Figure S7A), which is necessary for NK cell activation; furthermore, pre-treatment with VPA does not abrogate NK cell CD107 degranulation against melanoma targets (Figure S7B). Collectively, these data demonstrate that virus-activated NK cell effector function combined with HDACi-induced upregulation of NK cell-activating ligands could be used to potentiate the early, innate phase of OV-mediated anti-tumor immunity.

### HDACInhibition Enhances HSV^GM-CSF^-Mediated CTL Priming against Human Melanoma

**Figure 5:**
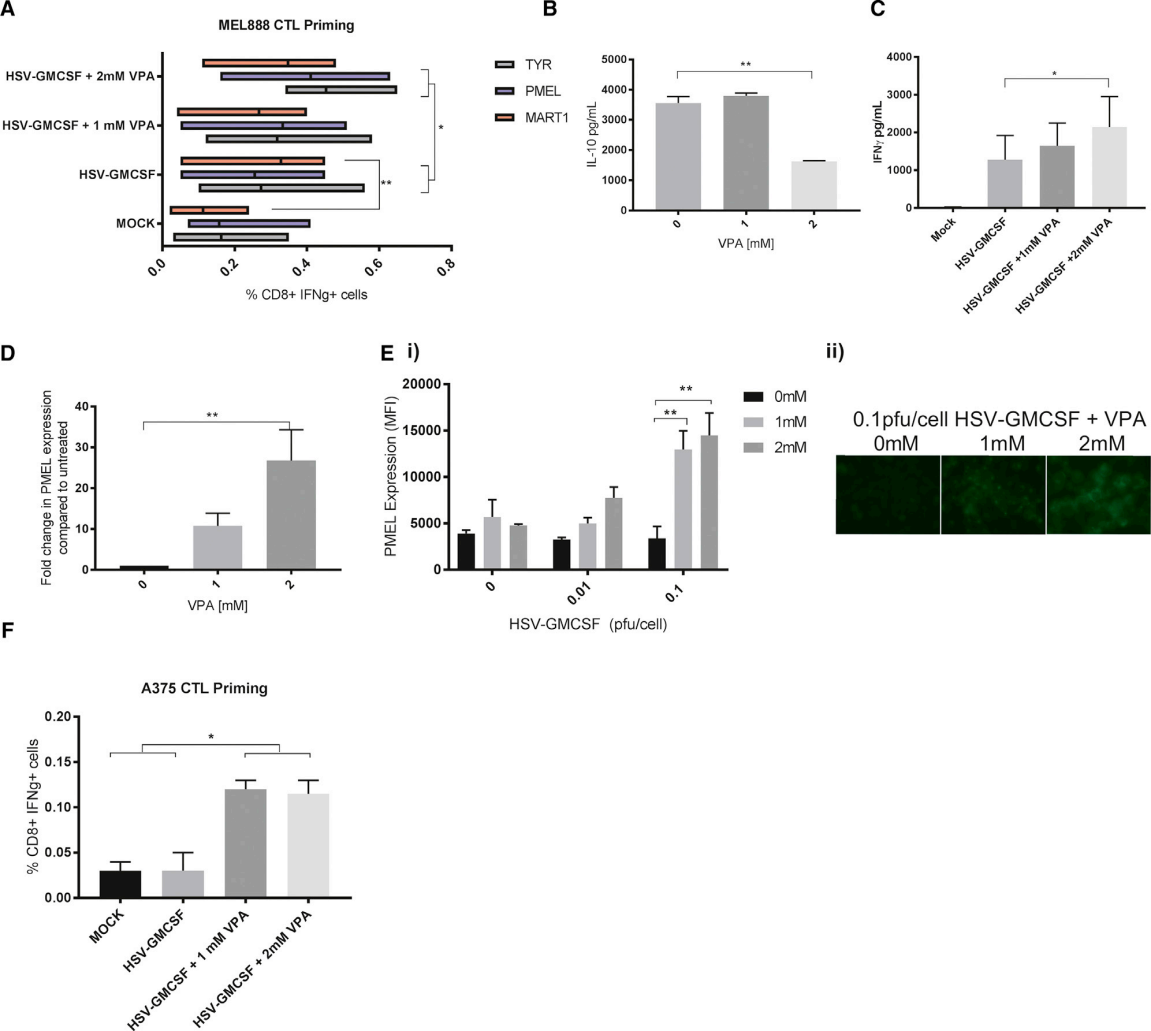
VPA Enhances HSV^GM-CSF^ CTL Responses against Melanoma (A–C) MEL888 cells were treated with the indicated doses of VPA for 24 h, followed by HSV^GM-CSF^ (0.1 PFUs/cell) and co-cultured with iDCs for 24 h; non-adherent cells (containing tumor-loaded APC) were removed and cultured with autologous PBMCs for 7 days. CTL cultures were re-stimulated appropriately and then used in TAA peptide recall assays. (A) The graph shows the mean (percent) of IFNγ^+^ CD8^+^ T cells following the indicated peptide recall (n = 4). (B) Cell-free supernatants from VPA-treated, HSV^GM-CSF^-infected MEL888 cells following co-culture with iDCs were collected, and the concentrations of IL-10 were determined by ELISA. The graph shows the mean + SEM (n = 3). (C) Cell-free supernatants from CTL cultures were collected on day 14, and the concentrations of IFNγ were determined by ELISA. The graph shows the mean + SEM (n = 4). (D–F) A375 cells were treated with the indicated doses of VPA for 24 h alone (D) or VPA followed by HSV^GM-CSF^ at the indicated doses (E and F). (D) *PMEL* mRNA expression levels were quantified by qRT-PCR relative to the *EF1α* housekeeping control following treatment with VPA for 24 h (n = 6). (E) Intracellular protein expression of PMEL was quantified by (i) flow cytometry (with or without VPA and/or with or without HSV^GM-CSF^; the graph shows the mean fluorescence intensity + SEM; n = 3) or (ii) immunofluorescence (with or without VPA and 0.1 PFUs/cell HSV^GM-CSF^). (F) A375 cells were treated with the indicated doses of VPA for 24 h, followed by HSV^GM-CSF^ (0.1 PFUs/cell) and co-cultured with iDCs for 24 h. Non-adherent cells (containing tumor-loaded APC) were removed and cultured with autologous PBMCs for 7 days. CTL cultures were re-stimulated appropriately and then used in a PMEL peptide recall assay. The graph shows the mean (percent) IFNγ^+^ CD8^+^ T cells + SEM (n = 2). Statistical significance is denoted by *p < 0.05 and **p < 0.01.

Having shown that HSV^GM-CSF^-treated melanoma cells can be used as an “antigen load” for iDCs to prime the generation of CTLs (Figure 1F), we went on to examine the consequences of HDAC inhibition for CTL priming. Moreover, to allow more complete characterization of the CTL response, we developed an immune readout component to allow tracking of T cell responses against a range of tumor-associated antigens (TAAs) without human leukocyte antigen (HLA) restriction. This adaptive immune readout involved pulsing autologous monocytes (capable of antigen processing and presentation) with 15-mer overlapping peptides of TAA (melanocyte protein PMEL [PMEL], tyrosinase [TYR], and melanoma antigen recognized by T cells 1 [MART-1/MELAN-A]) and co-culturing these with CTLs. TAA peptide recall responses by CTLs were then analyzed by flow cytometry to quantify intracellular IFNγ production. As shown in Figure 5A, HSV^GM-CSF^ infection of MEL888 cells enhanced the CD8 response against MEL888-expressed TAA, PMEL, MART-1, and TYR, with significant enhancement observed for MART-1 (p = 0.0028). Moreover, the quantity of TAA-specific responses measured against PMEL and TYR was significantly increased by co-treatment of MEL888 cells with 2 mM VPA and HSV^GM-CSF^ virus compared with HSV^GM-CSF^ alone. Interestingly, MEL888 cells treated with 2 mM VPA prior to HSV infection and then co-cultured with iDCs had significantly reduced levels of IL-10 in cell culture supernatants compared with virus-alone controls (Figure 5B), and higher concentrations of IFNγ were detected in CTL culture supernatants (Figure 5C); thus, the cytokine changes resulting from combination treatment may favor the generation of TAA-specific CTLs.

As well as addressing whether VPA could boost the CD8 response against TAAs expressed by melanoma cells, we also considered whether HDAC inhibition might alter the expression of TAA by melanoma cells and potentially broaden the range of antigens available for CTL priming. Initial studies demonstrated that VPA treatment of MEL888 cells did not increase PMEL, MART-1, or TYR expression at the protein level (data not shown). A375 cells do not express PMEL under normal growth conditions; however, following treatment with VPA or alternative HDACIs, significant increases in *PMEL* mRNA expression levels were detected (Figure 5D; Figure S8) Moreover, using flow cytometry and immunofluorescence techniques, we could detect PMEL protein expression following VPA/HSV^GM-CSF^ co-treatment but, interestingly, not following either treatment alone (Figures 5Ei and 5Eii, respectively). Following these observations, the ability of VPA to facilitate the generation of PMEL-specific CTLs, using A375 cells as the antigen load, was investigated. Figure 5F shows that only A375 cells treated with VPA/HSV^GM-CSF^, but not virus alone, were capable of generating PMEL-specific CTLs. Taken together, we have shown that HSV^GM-CSF^ infection supports human adaptive CTL priming against a range of melanoma-associated TAAs and that this priming is further increased by VPA, which can boost both the range and level of responses against targeted epitopes.

## Discussion

OVs represent a promising class of novel cytotoxic and immunogenic cancer therapy. Although T-Vec is the most clinically advanced agent, there are few pre-clinical data to inform its future development and optimal use, particularly in human systems. Despite its current application as an intratumoral treatment for melanoma, initial melanoma studies were restricted to testing of a single cell line for cytotoxicity *in vitro* only, using an early form of the virus that did not encode GM-CSF.^32^ Therefore, in the current study, we sought to address the role of both direct oncolysis and anti-tumor immunity (both innate and adaptive) in T-Vec efficacy using a closely related JS-1 strain of HSV-1 virus encoding GM-CSF, both alone and in combination with HDAC inhibition.

In our first experiments, we extended our previous studies using the dsRNA OV reovirus^22^, ^24^, ^25^, ^26^ to test the potential of the DNA virus HSV^GM-CSF^ to stimulate innate and adaptive anti-melanoma immunity (Figure 1). We found that (1) addition of HSV^GM-CSF^ to human PBMCs activated perforin and granzyme-mediated NK killing of melanoma targets, (2) HSV^GM-CSF^ induced maturation of iDCs, and (3) HSV^GM-CSF^ infection supported the generation of melanoma-specific CTLs. This ability of HSV^GM-CSF^ to activate innate and subsequent adaptive anti-tumor immunity supports its designation as an immunotherapeutic agent in humans. At present, the consequences of innate immune activation following administration of OVs remain controversial. For example, murine models suggest that the innate anti-viral NK cell response limits therapy by restricting direct tumor oncolysis^20^, ^31^, ^33^, ^34^ and viral replication and spread. Alternatively, NK cells have been shown to be essential for the success of a number of OVs across a range of pre-clinical models,^35^, ^36^, ^37^, ^38^, ^39^ suggesting that the innate response to the virus is critical for therapy. Furthermore, for other HSVs, experimental models have demonstrated the dependence of intratumoral HSV-1-induced melanoma therapy on NK cells,^40^ and studies have described the effectiveness of UV-inactivated HSV in stimulation of PBMCs to kill acute myeloid leukemia tumor cells in the absence of direct oncolysis.^41^ These lines of evidence support a positive role of the innate response in HSV therapy.

Currently, clinical use of T-Vec is restricted to intratumoral delivery; however, we know that the virus is subsequently released systemically because it can be detected in the circulation and in lymph nodes.^5^, ^10^ This viremia, which is consistent with transient flu-like symptoms and induction of an anti-viral antibody response, means that the virus has the potential to activate anti-tumor immunity systemically as well as locally in the tumor. Therefore, our study of the human innate effects of HSV^GM-CSF^ on PBMCs as well as on infected melanoma cells as the antigen load in a CTL priming assay remains clinically relevant. However, it is also important to note that HSV will initially engage an immunologically suppressive TME comprising Tregs, myeloid-derived suppressor cells (MDSCs), and M2-polarized macrophages; however, despite this, HSV has the capacity to modulate the TME^4^ to enrich levels of melanoma-specific effector T cells and decrease levels of Tregs at the site of tumor viral injection.^7^ Interestingly, HSV has been reported to inactivate MDSCs,^42^ and it is possible that HSV injection could increase NK cell infiltration at the tumor site, as described for alternative OVs,^43^ but this remains undescribed for T-Vec.

The work illustrated is especially important because the effects described here cannot be reliably modeled in murine systems. In particular, we tried testing HSV^GM-CSF^ alone or in combination with VPA in murine *in vitro* and *in vivo* systems but found that mouse tumor cells (e.g., B16 melanoma cells) were far more resistant to HSV^GM-CSF^ than human melanoma cells, and VPA was unable to increase NK cell-activating ligands on murine melanoma cells. No meaningful comparative results could be obtained in murine models, and we believe that this was due to major inherent differences between human melanoma models and murine immunocompetent models (in particular, only humans are natural hosts for type I HSV) rather than any lack of potential combination therapeutic benefit to patients.

In terms of the mechanisms by which HSV activates a human immune response, we found, as with reovirus,^24^ that production of type I IFNs, mediated by monocytes, was required (Figure 2). However, these innate responses are not identical for all OVs; for example, monocytes are not required for IFN production induced by ssRNA coxsackievirus (unpublished data), and the IFNγ and IL-29 secretion we observed in response to HSV^GM-CSF^ (Figure 2C) was not seen with reovirus.^22^, ^44^ The detailed mechanisms by which viral detector cells respond to ssRNA, dsRNA, and DNA viruses and how these shape the ensuing adaptive immune response are worthy of further study and are likely to inform the development of optimally immunogenic virotherapy, particularly as part of combination strategies. However, despite the clear role of monocytes in type I IFN production and subsequent NK cell activation, NK cells were still activated (although to a lesser extent) in the absence of monocytes, and low-level IFNα production was still observed. Therefore, it is possible that alternative mechanisms may also be involved in the detection of HSV; for example, plasmacytoid DCs have also been reported to play a role in regulating anti-HSV immune responses.^45^, ^46^, ^47^

Among the various immunomodulatory strategies tested in combination with OVs, HDACIs have been explored as a means to enhance virus-mediated oncolysis via suppression of the tumor cell anti-viral IFN response following infection.^16^, ^31^ However, HDAC inhibition has a wide range of consequences, and a recent study demonstrated, using cDNA arrays, that the expression of 10%–20% of genes was altered following treatment with HDACIs.^48^ We found that pre-treatment of human melanoma cell lines with VPA increased cytotoxicity, GM-CSF secretion, and viral replication upon infection with HSV^GM-CSF^ (Figure 3), although no effect of HDACi on IFN production or expression of IFN-stimulated genes by tumor cells was observed following virus infection (data not shown). In fact, we were unable to detect IFNα, IFNβ, IL-28a, or IL-28b/IL-29 by ELISA following HSV^GM-CSF^, and gene expression results confirmed that viral treatment did not induce an IFN signaling cascade (data not shown). Moreover, we also explored the possibility that VPA could alter the surface expression of the HSV receptors HVEM and Nectin 1 or alter NF-κB signaling;^49^ no changes were observed following VPA treatment (data not shown). Currently the mechanisms responsible for enhanced HSV^GM-^CSF-induced direct oncolysis following VPA treatment remain to be fully elucidated; however, an alternative mechanism could be that VPA alteration of chromatin structure prevents HSV^GM-CSF^ from “hiding” within DNA, making it more accessible for viral replication.^50^

Perhaps more importantly, from an immune perspective, we found that HDACIs upregulated expression of NKG2D ligands on melanoma cells, leading to increased NK cell killing by HSV^GM-CSF^-activated PBMCs (Figure 4). Furthermore, in a novel assay that quantified non-HLA-restricted anti-TAA CTL priming, addition of HDACIs to HSV^GM-CSF^ treatment of melanoma cells enhanced both the magnitude and range of TAAs expressed by tumor cells as targets for CD8 T cell recognition (Figure 5). Although it was unexpected that PMEL expression was only observed at the protein level upon co-treatment of VPA and HSV^GM-CSF^, because HSV infection is usually associated with “shutoff” of host protein translation to limit viral detection and allow propagation,^51^, ^52^ the balance between histone acetylation or deacetylation is important for HSV propagation.^53^ Therefore, it is possible that HDAC inhibition by VPA allows transcription of PMEL mRNA for subsequent processing in HSV-infected cells, where HSV will employ a range of strategies to stimulate viral protein synthesis, including enhancement of translation initiation and prevention of translation shutdown following cell stress.^52^ We are currently investigating whether the expanded range of antigens recognized by primed CTLs in combination VPA/HSV^GM-CSF^ treatment extends to neoantigens as well as the shared, non-mutated TAAs we have tracked here and whether this is reflected in the T cell receptor repertoire, which develops over time. Importantly, in terms of clinical application of VPA with HSV^GM-CSF^, the doses of VPA used to potentiate direct oncolysis and anti-tumor immunity would be clinically achievable, with current therapeutic ranges for epilepsy and mania ranging between 20–125 mg/L (0.15–0.87 mM), only marginally lower than the doses utilized in this study; higher serum concentrations are clinically achievable with appropriate monitoring for additional toxicity.^54^, ^55^

In summary, we have shown, using clinically relevant human pre-clinical models of innate and adaptive anti-tumor immune priming, that HSV^GM-CSF^ is capable of activating an anti-melanoma immune response and that the cytotoxicity and immunogenicity of the currently most clinically advanced class of OV is further boosted by combination with HDAC inhibition. These data provide a platform to explore further OV and immunotherapy combination strategies in human pre-clinical systems and support incorporation of clinical HDACIs into future HSV-based OV clinical trials.

## Materials and Methods

### Cell Culture and Reagents

The A375, MeWo, and Vero cell lines were purchased from the ATCC and authenticated using short tandem repeat (STR) profiling and comparison with the DSMZ database. MEL888^56^ and MM96^57^ cells were obtained from the Cancer Research UK cell bank, and MCF-7 cells were kindly provided by M. Muthana (Department of Oncology and Metabolism, University of Sheffield). In the absence of a reference profile in the DSMZ database, cell lines were shown to have an original STR profile that was distinct from all other cell lines in the database. HFFs were also purchased from the ATCC. All cell lines and HFFs were grown in glutamine-containing DMEM (Sigma-Aldrich) supplemented with 10% fetal calf serum (FCS) (v/v) (Biosera). All cell lines were routinely checked for mycoplasma and were free from contamination.

PBMCs were isolated from healthy donor volunteers or melanoma patients after written informed consent was obtained in accordance with local institutional ethics and review approval (06/Q1206/106). PBMCs were isolated from whole blood by density gradient centrifugation on Lymphoprep (Aldere) and cultured at 2 × 10^6^ cells/mL in glutamine-containing RPMI medium (Sigma-Aldrich) supplemented with 10% FCS (v/v). Where indicated, NK cells were freshly isolated, or CD14^+^ cells were removed or isolated from PBMCs using MACS isolation procedures, following the manufacturer’s protocols (Miltenyi Biotec). iDCs were generated by culturing CD14^+^ cells in glutamine-containing RPMI medium supplemented with 10% FCS (v/v), recombinant human IL-4 (500 IU/mL), and GM-CSF (800 IU/mL) (both from R&D Systems) at a cell density of 1–2 × 10^6^ cells/mL for 5 days. CTLs were cultured at 4 × 10^6^ cells/mL in glutamine-containing RPMI medium supplemented with 7.5% (v/v) human AB serum, 1 mM sodium pyruvate, 1 mM HEPES, 1% (v/v) non-essential amino acids, 20 μM 2β-mercaptoethanol (all from Sigma-Aldrich) and recombinant human IL-7 (5 ng/mL) (Miltenyi Biotec). VPA (Sigma-Aldrich) was added to cell cultures at 0, 1, or 2 mM for the indicated durations.

### Viruses

JS1 34.5-human GM-CSF (hGM-CSF) 47-pA^+^ (HSV^GM-CSF^) was kindly provided by Amgen, and the virus titer was determined by a standard plaque assay on Vero cells. The JS1 34.5-hGM-CSF 47-pA^+^ used in these studies differs from the clinical agent tamilogene laherparepvec in that the US11 gene is not under control of the ICP47 intermediate-early promoter because of the addition of a poly(A) sequence between the promotor and the US11 coding sequence.

### HSV^GM-CSF^ Replication

Cells were treated with HSV^GM-CSF^ alone, HSV^GM-CSF^ following 24-h pre-treatment with VPA, or VPA/HSV^GM-CSF^ simultaneously. Cells and supernatants were harvested and subjected to three rounds of freezing and thawing using a 37°C water bath and methanol and dry ice. HSV^GM-CSF^ concentration was determined by standard plaque assay on Vero cells, and the fold increase in virus titer was determined by comparison with the input virus.

### MTT Cell Viability

Melanoma cell lines were seeded at 8 × 10^3^ cells/well into 96-well plates and left to adhere overnight. Cells were treated with HSV^GM-CSF^ at the indicated doses for 48 h (with or without pre-treatment with VPA for 24 h). 20 μL MTT (5 mg/mL, Sigma-Aldrich) was added to cells 4 h prior to the end of the incubation period before the tissue culture supernatant was removed, and cells were solubilized using 150 μL DMSO (Sigma-Aldrich). Optical density absorbance readings were determined using a Thermo Multiskan EX plate reader (Thermo Fisher Scientific) at 540 nm absorbance.

### ELISA

The production of human GM-CSF, IFNα (both from Mabtech), IFNβ (PBL Interferon Source), IL-10, TNF-α, IFNγ (all from BD Pharmingen) and IL-29 (R&D Systems) in cell-free supernatant was determined using matched-paired antibodies according to the manufacturers’ instructions. Optical density absorbance readings were determined using a Thermo Multiskan EX plate reader (Thermo Fisher Scientific) at 405 nm absorbance.

### Cell Surface Phenotyping

Cell surface expression of the indicated markers was quantified by flow cytometry. Briefly, cells were harvested, washed in fluorescence-activated cell sorting (FACS) buffer (PBS, 1% [v/v] FCS, and 0.1% [w/v] sodium azide), and incubated for 30 min at 4°C with specific antibodies or matching isotype controls. Cells were washed with FACS buffer and then fixed with 1% paraformaldehyde (PFA) (1% [w/v] PFA in PBS) and stored at 4°C prior to acquisition. Flow cytometry analysis was performed either using a FACSCalibur (and analysis was carried out using Cell Quest Pro software; BD Biosciences), an Attune flow cytometer (Life Technologies, with analysis performed on its accompanying software), or a CytoFLEX S (Beckman Coulter; analysis carried out using CytExpert software).

### Intracellular Staining

Cells were cell surface-stained and fixed overnight with 1% PFA prior to permeabilization with 0.3% saponin (Sigma-Aldrich) for 15 min at 4°C. Cells were washed with 0.1% saponin and incubated with specific antibodies or matched isotype controls for 30 min at 4°C. If the primary antibody was fluorescently-unconjugated, then cells were incubated with a matched fluorescently conjugated antibody for 30 min at 4°C. Cells were washed with PBS, and flow cytometry analysis was performed immediately using a CytoFLEX S.

### Flow Cytometry Antibodies

The following flow cytometry antibodies were used: CD11c APC-Vio770 (MJ4-27G12, Miltenyi Biotec), CD14-PerCP (TUK4, Miltenyi Biotec), CD86-phycoerythrin (PE)-Cy7 (2331, BD Biosciences), CD80-PE (L307.4, BD Biosciences), HLA-ABC-VioBlue (REA230, Miltenyi Biotec), HLA-DR/DP/DQ-fluorescein isothiocyanate (FITC) (Tu39, BD Biosciences), CD3-PerCP (SK7, BD Biosciences), CD56-PE (B159, BD Biosciences), CD8-APC (RPA-T8, BD Biosciences) CD107a-FITC (H4A3, BD Biosciences), CD107b-FITC (H4B4, BD Biosciences), CD69-FITC (FN50, BD Biosciences), MICA/B-PE (6D4, BD Biosciences), ULBP2/5/6 (FAB1298p, R&D Systems), IFNγ-BV421 (4S.B3, BD Biosciences), mouse immunoglobulin G1 (IgG1) κ isotype control (PE/FITC/PerCP/PE-Cy7/APC; MOPC-21, BD Biosciences), mouse IgG2a κ isotype control (FITC/PE; G155-178, BD Biosciences), and REA Control-VioBlue (REA293, Miltenyi Biotec).

### CD107a/b NK Cell Degranulation Assay

Healthy donor or melanoma patient PBMCs were treated with HSV^GM-CSF^ overnight and co-cultured with melanoma cell targets at a 10:1 ratio for 1 h at 37°C. 10 μg/mL Brefeldin A (BioLegend), anti-CD107a/b, anti-CD3, and anti-CD56 were added for a further 4 h at 37°C before cells were washed with FACS buffer and fixed using 1% PFA. Flow cytometry analysis was performed using either the Attune or CytoFLEX S flow cytometers.

### Flow Cytometry Killing Assay

Healthy donor PBMCs were activated with HSV^GM-CSF^ overnight at the indicated concentrations, and their ability to kill melanoma cell targets (with or without VPA treatment) stained with Cell Tracker Green (Molecular Probes) was determined using standard 5-h co-culture. Co-cultures were washed and stained for viability using a live-dead fixable dead cell stain (Thermo Fisher Scientific) before analysis using an Attune flow cytometer.

### Neutralization of Type I IFNs

PBMCs were treated with HSV^GM-CSF^ overnight in the presence or absence of neutralizing antibodies (IFN Block, PBL Interferon Source) or an isotype control (IFN Isotype, R&D Systems). The IFN block consisted of sheep polyclonal anti-human IFN-α, sheep polyclonal anti-human IFN-β (both used at 1.5%), and mouse monoclonal anti-human IFN-α/β receptor chain 2 (used at 2.5%), as described previously.^22^ The isotype control consisted of sheep serum (Sigma-Aldrich) used at 3% and mouse IgG2a used at 2.5%. PBMCs were then washed and used in CD107 degranulation assays, ^51^Cr cytotoxicity assays, or stained for cell surface expression of CD69 as described above.

### Real-Time qPCR

Total RNA from cells was isolated using TRiZol (Invitrogen), and 1 μg of RNA was used to synthesize cDNA using Maxima Reverse Transcriptase (Thermo Fisher Scientific) according to the manufacturer’s instructions. Real-time qPCR was carried out with SYBR Green mix (Applied Biosciences) using a QuantStudio5 real-time PCR system (Thermo Fisher Scientific). Primer sequences were as follows: *PMEL*-F (5′-TATCATGCCTGTGCCTGGGA-3′) and *PMEL*-R (5′-GGGGTACGGAGAAGTCTTGC-3′) for *PMEL* and *EIFA*-F (5′-GATTACAGGGACATCTCAAGGCG-3′) and *EIFA*-R (5′-TATCTCTTCTGGCTGTAGGGTGG-3′) for the *EIFA* housekeeping control.

### Immunofluorescence

Cells were fixed with 4% PFA and permeabilized with 0.1% Triton X-100 (Sigma-Aldrich). Samples were incubated with anti-melanoma PMEL antibody (gp100) at a dilution of 1/250 (EP4863 [2], Abcam), followed by goat anti-rabbit IgG (Alexa Fluor 488, Abcam) secondary antibody, following the manufacturer’s instructions. Cells were then imaged using the EVOS imaging system (Thermo Fisher Scientific).

### Cytotoxic T Cell Priming Assay

Melanoma cells were treated with or without VPA 24 h prior to addition of HSV^GM-CSF^ and iDCs. Non-adherent cells (iDCs loaded with TAA) were removed 24 h after addition of HSV^GM-CSF^ and cultured with autologous PBMCs for 7 days. CTLs were re-stimulated (as previously) and cultured for a further 7 days. Primed CTLs were then harvested and used in a 4-h ^51^Cr release assay or peptide recall assays.

### ^51^Cr Release Assay

HSV^GM-CSF^-treated PBMCs (with or without NK cell depletion) or CTLs were co-cultured with ^51^Cr (PerkinElmer)-labeled MEL888, A375, MeWo, or MCF-7 cells at different effector:target (E:T) ratios for 4 h (with or without 2 mM EGTA where indicated). Cells were then pelleted by centrifugation, and 50 μL of supernatant was transferred to scintillation plates (PerkinElmer) prior to analysis using a Wallac Jet 1459 Microbeta scintillation counter and Microbeta Windows software (PerkinElmer). Tumor cell percentage lysis was determined using the following calculation.

%lysis=(SampleCPM−SpontaneousCPM)/(MaximumCPM−SpontaneousCPM)×100

### Peptide Recall Assay

To measure peptide-specific CTL responses, autologous CD14^+^ cells were incubated with the PMEL, TYR, or MART-1/MELAN-A PepTivator peptide pools (15-mer peptide sequences with 11-amino acid overlap, Miltenyi Biotec) for 60 min at 37°C according to the manufacturer’s instructions. Autologous CD14^+^ cells with or without peptide labeling were then co-cultured with CTLs for 60 min at 37°C before addition of Brefeldin A (1:1,000, BioLegend) and an anti-CD8-APC antibody for identification of CTLs. CTLs were then incubated for a further 4 h at 37°C, and cells were fixed prior to intracellular IFNγ staining and acquisition and analysis by flow cytometry.

### Statistical Significance

Statistical analysis was carried out with the GraphPad Prism software. Statistical differences among groups were determined using Student’s t test, one-way ANOVA, or two-way ANOVA. The criterion for statistical significance was p value of less than 0.05.

## Author Contributions

Conceptualization, F.E.-M. and A.A.M.; Methodology, V.A.J.; Investigation, V.A.J., G.B.S., A.M.S.R., K.J.S., G.M., and K.R.; Resources, B.K., O.D., H.P., D.D., and A.S.; Writing – Original Draft, F.E.-M., A.A.M., and V.A.J.; Writing – Review & Editing, K.J.S., K.J.H., H.P., and R.G.V.; Supervision, F.E.-M., A.A.M., and V.A.J.; Funding Acquisition, A.A.M.

## Conflicts of Interest

K.J.H. and A.A.M. received consultancy fees and travel support from Amgen Inc.

## Supplemental Information

Document S1. Figures S1–S8

Document S2. Article plus Supplemental Information
