# Supplementary material for: Potentiating Oncolytic Virus-Induced Immune-Mediated Tumor Cell Killing Using Histone Deacetylase Inhibition
Source: Mol Ther. 2019 Apr 14;27(6):1139–52. doi: 10.1016/j.ymthe.2019.04.008 (PMC6554638; doi:10.1016/j.ymthe.2019.04.008)
Supplement: Document S1. Figures S1–S8 [file mmc1.pdf]

YMTHE, Volume *27*

Supplemental Information

Potentiating Oncolytic Virus-Induced Immune-Mediated Tumor Cell Killing Using Histone Deacetylase Inhibition

Victoria A. Jennings, Gina B. Scott, Ailsa M.S. Rose, Karen J. Scott, Gemma Migneco, Brian Keller, Katrina Reilly, Oliver Donnelly, Howard Peach, Donald Dewar, Kevin J. Harrington, Hardev Pandha, Adel Samson, Richard G. Vile, Alan A. Melcher, and Fiona Errington-Mais

Supplementary Figure 1

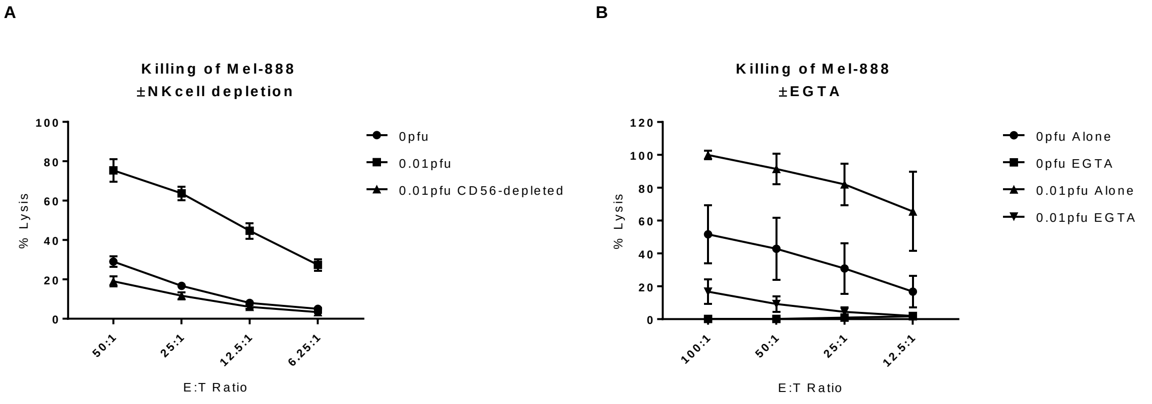

Supplementary Figure 1. NK cells are responsible for lysis of MEL888 target cells. A. PBMC were either left untreated (0 pfu/cell) or treated with 0.01pfu/cell HSV$^{GM\text{-}CSF}$ overnight, before PBMC were then either left intact (0.01pfu) or NK cells were depleted using CD56^{+} MACS selection (0.01pfu CD56-depleted) prior to ^{51}Cr release assays. The mean percentage lysis of ^{51}Cr-labelled MEL888 tumor cell targets is shown (n=3; ±SEM). **B**. PBMC were either left untreated (0 pfu/cell) or treated with 0.01pfu/cell HSV$^{GM\text{-}CSF}$ overnight. Percentage lysis of ^{51}Cr-labelled MEL888 targets was determined in the presence or absence of 2mM EGTA (n=2, ±SEM).

Supplementary Figure 2

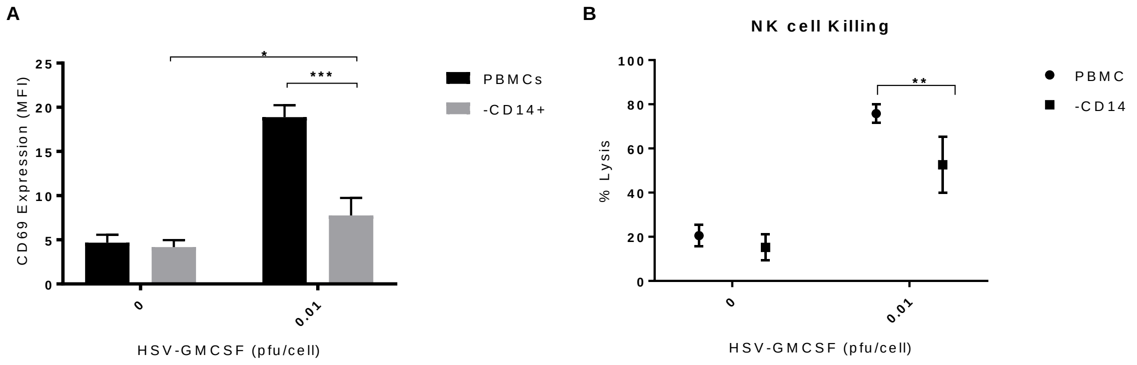

Supplementary Figure 2. Depletion of CD14+monocytes from PBMC decreases NK cell activation. A. Heathy donor PBMC (±$CD14^+$ monocyte depletion) were treated with HSV^{GM-CSF} overnight and the expression of CD69 on NK cells was determined by flow cytometry. Data shows the average mean fluorescence intensity of CD69 expression on NK cells (n=4, +SEM). **B**. Heathy donor PBMC (±$CD14^+$ monocyte depletion) were treated with HSV^{GM-CSF} overnight, co-cultured with MEL888 cells for 4hrs, and target cell lysis was determined by ^{51}Cr release. Data shows the average percentage tumor cell lysis (n=4, ±SEM).

Supplementary Figure 3

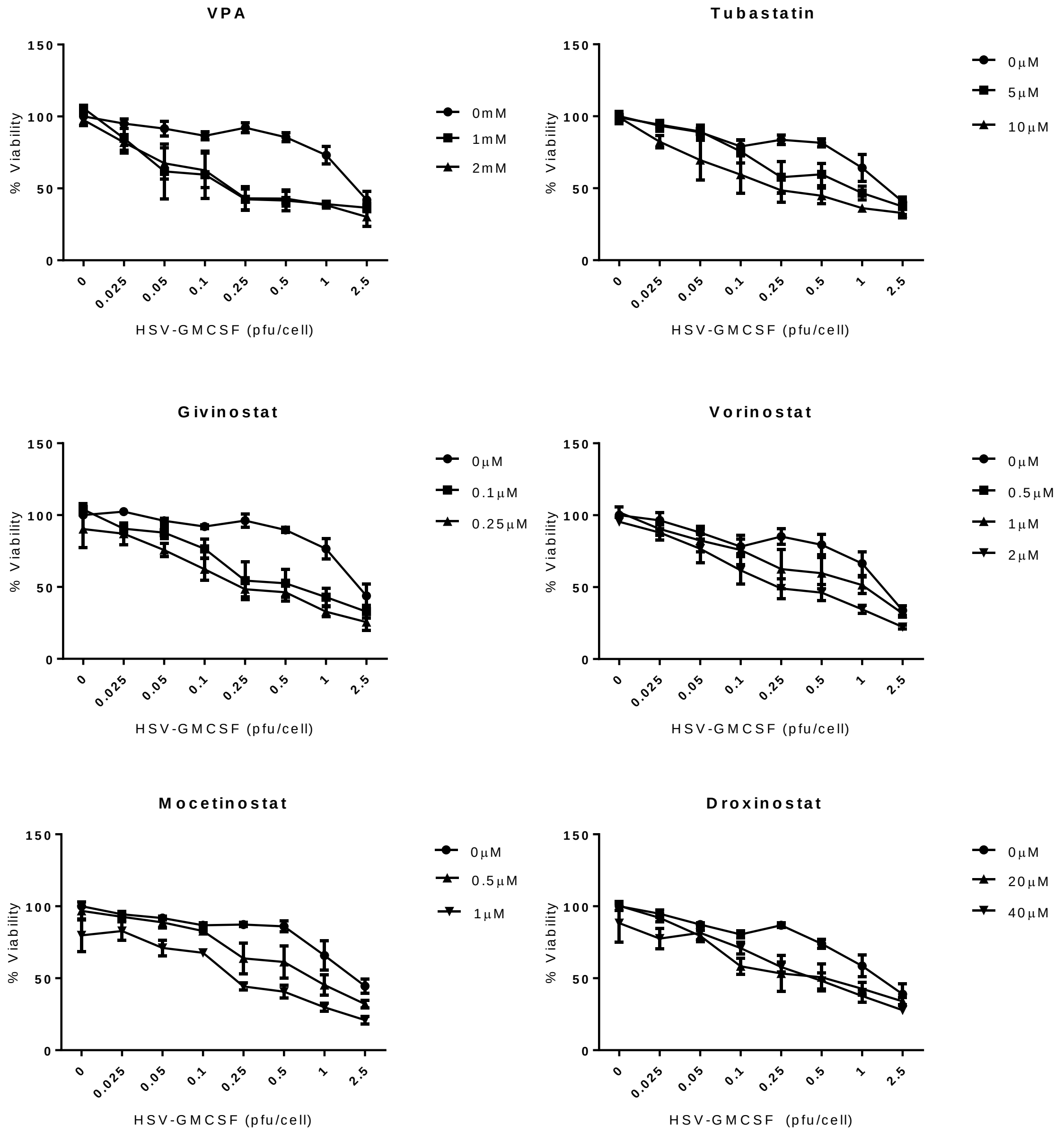

Supplementary Figure 3. Multiple HDACi enhance HSV$^{GM\text{-}CSF}$-induced cytotoxicity. Melanoma cell lines were seeded and treated with a range of HDACi at sub toxic doses for 24hrs, prior to the addition of HSV$^{GM\text{-}CSF}$ at concentrations ranging from 0 to 2.5pfu/cell. Cells were left for a further 48hrs and cell viability was determined by MTT assay. Data shown is the average of at least three independent experiments, ±SEM.

Supplementary Figure 4

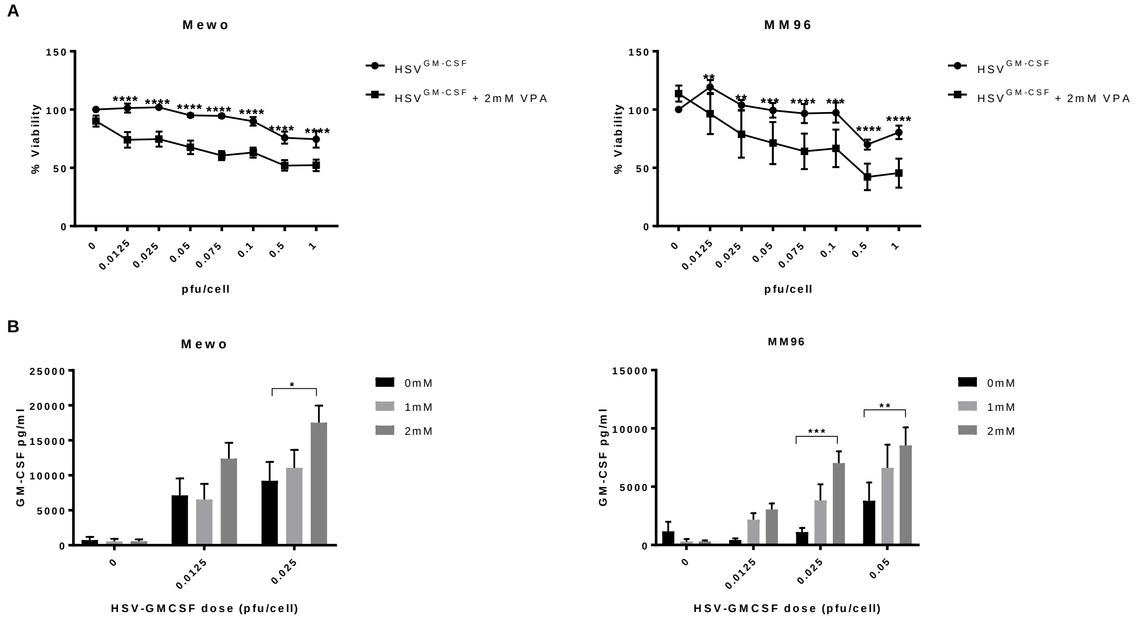

Supplementary Figure 4: VPA enhances HSV^{GMCSF} cytotoxicity and transgene expression. A. Melanoma cell lines (MeWo and MM96) were seeded and treated with VPA for 24hrs prior to the addition of HSV$^{GM\text{-}CSF}$ at concentrations ranging from 0 to 1pfu/cell. Cells were left for a further 48hrs and cell viability was determined by MTT assay. Data shown is the average of at least four independent experiments ±SEM. **B**. VPA-treated cells were treated with HSV$^{GM\text{-}CSF}$ for 24hrs and GM-CSF production was determined by ELISA. Data shows the mean of at least five independent experiments +SEM. Statistical significance is denoted by *p<0.05, **p<0.01, p***< 0.005, ****p<0.0001.

Supplementary Figure 5

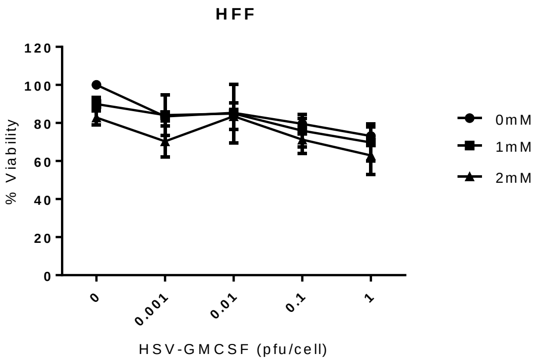

Supplementary Figure 5. VPA does not enhance HSV$^{GM\text{-}CSF}$ cytotoxicity in normal HFF. HFF were seeded and treated with 0, 1 or 2mM VPA for 24hrs, prior to the addition of HSV$^{GM\text{-}CSF}$ at concentrations ranging from 0 to 1pfu/cell. Cells were left for a further 48hrs and cell viability was determined by MTT assay (n=3, ±SEM).

Supplementary Figure 6

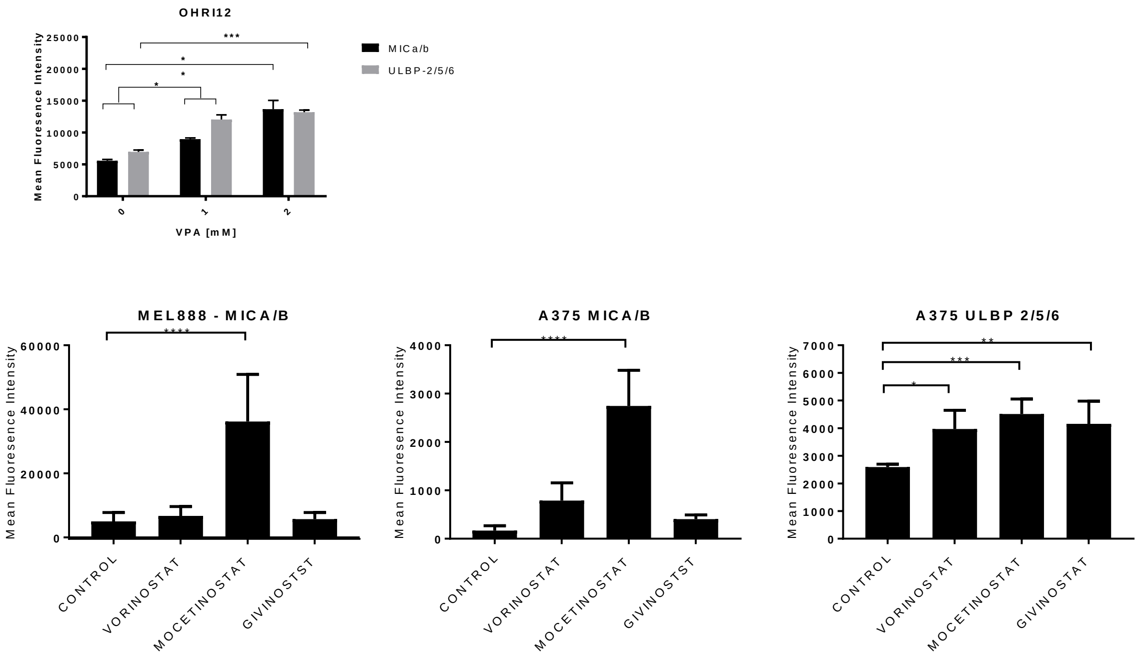

Supplementary Figure 6: VPA up-regulates NK cell activatory ligands on primary melanoma cells and alternative HDACi modulate MICA/B and ULBP2/5/6 expression. A. Expression of NK ligands (MICA/B and ULBP2/5/6) on the surface of primary melanoma cells (OHRI12) was determined by flow cytometry. Cells were treated with VPA at indicated doses for 48hr (n=3, +SEM). **B**. MEL888 and A375 cells were treated with 2mM VPA, 2µM vorinostat, 1µM mocetinostat and 0.2µM givinostat for 48hrs and the expression of MICA/B and ULBP2/5/6 was determined by flow cytometry (n=3, +SEM).

Supplementary Figure 7

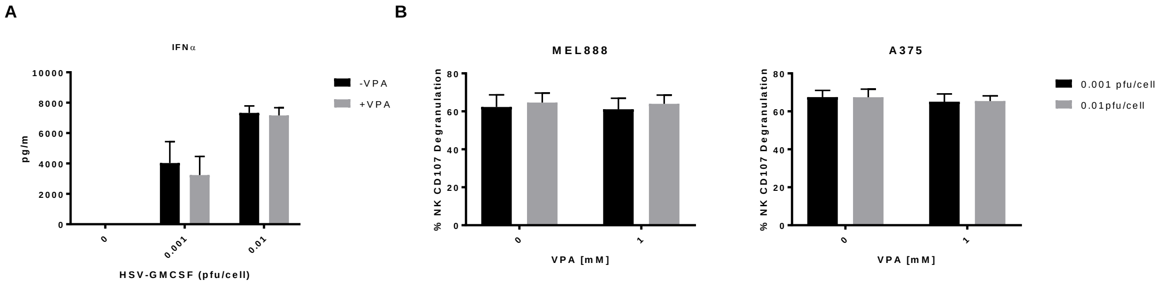

Supplementary Figure 7. Pre-treatment of PBMC with VPA does not prevent IFNα production or NK cell activation. A. IFNα production from PBMC pre-treated ±VPA for 4 hrs followed by HSV^{GM-CSF} treatment (0, 0.01 and 0.001pfu/cell) overnight was determined by ELISA (n=3, +SEM); un-bound VPA was removed prior to addition of HSV^{GM-CSF}. **B**. Healthy donor PBMC were treated ±VPA for 4 hours followed by HSV^{GM-CSF} treatment (0.01 and 0.001pfu/cell) overnight. PBMC were co-cultured with melanoma targets and NK cell ($CD56^+$/$CD3^-$) CD107 degranulation was determined by flow cytometry. Data shows the mean percentage of NK cells expressing CD107 after co-culture with MEL888 and A375 cell targets (n=3, +SEM).

Supplementary Figure 8

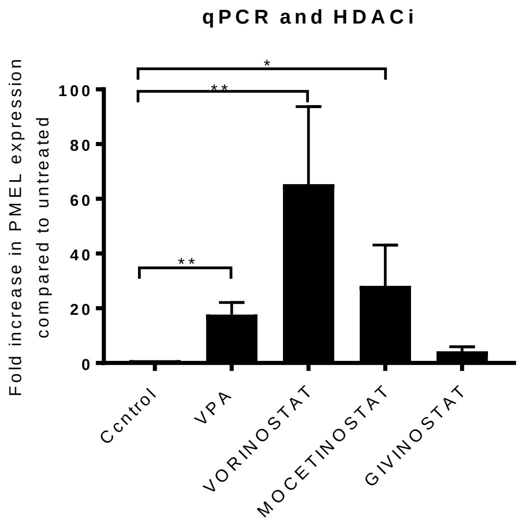

Supplementary Figure 8. Alternative HDACi up-regulate PMEL TAA expression. PMEL mRNA expression levels in A375 cells were quantified by qRT-PCR relative to EF1α housekeeping control following treatment with 2mM VPA (n=6, +SEM), 2µM vorinostat, 1µM mocetinostat and 0.1µM givinostat for 48hrs (n=3, +SEM).
